# Site-of-Metabolism Prediction with Aleatoric and Epistemic Uncertainty Quantification

**DOI:** 10.1021/acs.jcim.5c00762

**Published:** 2025-08-06

**Authors:** Roxane Axel Jacob, Oliver Wieder, Ya Chen, Angelica Mazzolari, Andreas Bergner, Klaus-Juergen Schleifer, Johannes Kirchmair

**Affiliations:** † Department of Pharmaceutical Sciences, Division of Pharmaceutical Chemistry, Faculty of Life Sciences, 27258 University of Vienna, Josef-Holaubek-Platz 2, Vienna 1090, Austria; ‡ Christian Doppler Laboratory for Molecular Informatics in the Biosciences, Department of Pharmaceutical Sciences, University of Vienna, Josef-Holaubek-Platz 2, Vienna 1090, Austria; § Dipartimento di Scienze Farmaceutiche, 60232 Universita degli Studi di Milano, Milano I-20133, Italy; ∥ Global Medicinal Chemistry, Boehringer Ingelheim RCV GmbH & Co KG, Vienna 1120, Austria; ⊥ 5184 BASF SE, Ludwigshafen am Rhein 67063, Germany; # Vienna Doctoral School of Pharmaceutical, Nutritional and Sport Sciences, University of Vienna, Josef-Holaubek-Platz 2, Vienna 1090, Austria

## Abstract

In silico metabolism prediction models have become indispensable tools to optimize the metabolic properties of xenobiotics while preserving their intended biological activity. Among these, site-of-metabolism (SOM) prediction models are particularly valuable for pinpointing metabolically labile atomic positions. However, the practical utility of these models depends not only on their ability to deliver accurate predictions but also on their capacity to provide reliable estimates of predictive uncertainty. In this work, we introduce aweSOM, a graph neural network (GNN)-based SOM prediction model that leverages deep ensembling to model the total predictive accuracy and partition it into its aleatoric and epistemic components. We conduct a comprehensive evaluation of aweSOM’s uncertainty estimates on a high-quality data set, identifying key challenges that currently constrain the performance of SOM prediction models. Based on these findings, we propose actionable insights to drive progress in the field of metabolism prediction.

## Graphical abstract

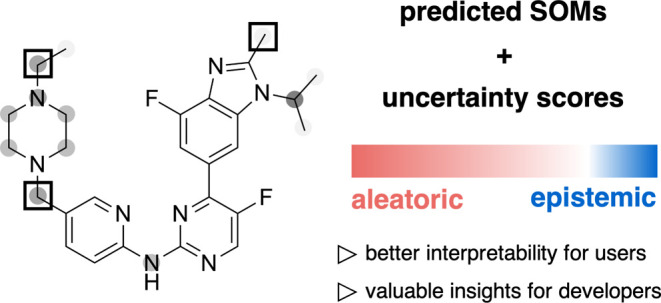

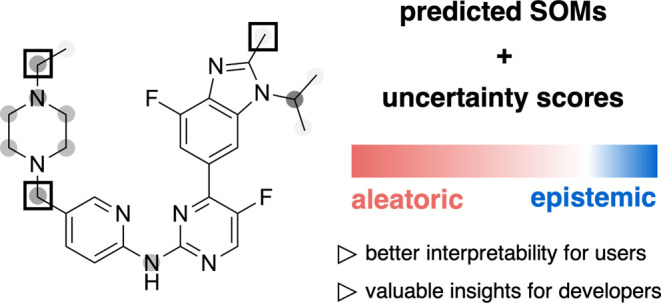

## 1 Introduction

Metabolic reactions significantly impact the efficacy and safety of agrochemicals, cosmetics, pharmaceuticals, and other xenobiotic compounds.^1^ While the majority of these reactions facilitate the detoxification and excretion of small organic molecules,^2^ a subset produces reactive intermediates or metabolites that may disrupt essential physiological functions.^2^ Additionally, the premature deactivation of bioactive compounds by xenobiotic-metabolizing enzymes poses a considerable challenge for the agrochemical and pharmaceutical industries.^2^ Thus, optimizing the metabolic properties of xenobiotics while preserving their intended biological functions remains a critical objective in the development of functional small organic molecules.

In recent years, *in silico* models for predicting xenobiotic metabolism have gained traction as valuable tools for medicinal chemists seeking to optimize metabolic properties.^3–9^ One of the most prominent applications of such models is the prediction of sites-of-metabolism (SOMs)—the specific atoms where metabolic reactions are initiated. Accurate identification of SOMs helps chemists enhance a compound’s metabolic stability while retaining its intended biological activity. Furthermore, SOM predictors often serve as integral components of metabolite structure prediction tools, supporting the identification and ranking of likely metabolites.^10–12^

Current SOM prediction models employ diverse methodologies, including machine learning algorithms, quantum chemical methods, molecular interaction fields, and docking-based approaches. For example, RS-predictor^13–15^ combines topological descriptors with precomputed, density functional theory (DFT)-derived transition state energies to predict cytochrome P450 (CYP)-mediated SOMs. XenoSite^16–22^ is a collection of neural network models designed to predict SOMs associated with phase I metabolism and UDP-glucuronosyl-transferases (UGT). SOMP^23^ uses a Bayesian approach to predict SOMs for five CYP isoforms and UGT. More recently, GNN-SOM^24^ was introduced as the first graph neural network (GNN)-based SOM predictor. FAME 3,^25^ a random forest-based model developed by some of the authors of this manuscript, distinguishes itself through its extensive coverage of both phase I and phase II biotransformation while achieving state-of-the-art classification accuracy and ranking performance.

Despite these advancements, SOM predictors remain constrained by the limited availability and variable quality of training data. Challenges such as biological variability, operational inconsistencies, the transient nature of reactive metabolites, difficulties in structural determination, and inconsistent data curation introduce varying degrees of uncertainty into the underlying data, which, in turn, propagate to model predictions. Addressing these limitations requires defining the applicability domain of SOM predictors and incorporating detailed uncertainty estimates to enhance their reliability.

Among existing SOM predictors, only FAME 3 provides an applicability score (FAMEscore), which quantifies prediction reliability based on the average Tanimoto distance between an atom of interest and its three nearest neighbors in the training data.^25^ However, this approach does not account for the number of similar atoms in the training set or the consistency of their labels, limiting its effectiveness as a measure of uncertainty.

Additionally, neural network-based predictors such as GNN-SOM and XenoSite generate classification outputs that cannot be directly interpreted as confidence scores.^26^ In well-calibrated binary classifiers, uncertainty can be inferred from output probabilities relative to the decision threshold. For instance, with a threshold of 0.5, a prediction of 0.6 indicates greater uncertainty than one of 0.9. However, only FAME 3 and GNN-SOM report per-atom probability scores, and neither addresses model calibration. Furthermore, while probabilistic outputs provide a measure of overall predictive uncertainty, they do not elucidate its underlying causes.

To overcome these limitations, more sophisticated uncertainty quantification (UQ) techniques are required. In line with this, recent studies^27–30^ have explored UQ in molecular property prediction and design, comparing approximation methods to Bayesian inference, such as mean-variance estimation, deep ensembles, Monte Carlo (MC) dropout, stochastic weight averaging, stochastic gradient Langevin dynamics, and Bayes by Backprop, to estimate predictive uncertainty and to distinguish between aleatoric and epistemic uncertainties.

Aleatoric uncertainty, also known as statistical or stochastic uncertainty, arises from inherent noise in the data and can only be reduced by improving data quality through repeated measurements or error correction.^31,32^ Epistemic uncertainty stems from a lack of knowledge about how best to model the underlying task and can, in principle, be reduced with additional information.^31,32^ It is further divided into model bias, or model uncertainty, and model variance, also known as approximation uncertainty. Model bias reflects the uncertainty introduced by assumptions, simplifications, or limitations in the model’s architecture and can usually be omitted when working within a specific predictive setting. Model variance relates to ambiguities in the model’s learned parameters after its architecture has been selected and is expected to disappear in the limit of infinite data.^31,32^

Existing UQ studies in molecular property prediction primarily address molecule-level regression tasks^27,33–36^ with several demonstrating that Bayesian inference enhances prediction quality while providing informative uncertainty estimates.^27,33,36,37^ However, no approach to date integrates uncertainty decomposition into atom-level classification tasks, such as SOM prediction.

In this work, we introduce a GNN-based SOM predictor, aweSOM, which leverages the deep ensemble method^38^ to estimate both aleatoric and epistemic uncertainty. We demonstrate that aweSOM achieves state-of-the-art performance for both phase I and phase II metabolism while offering three key advantages: (1) enabling users to assess prediction reliability more effectively, (2) aiding model developers in identifying sources of error and refining predictive models, and (3) highlighting problematic compounds and structural motifs, thereby guiding data-driven improvements in xenobiotic metabolism prediction.

## 2 Materials and Methods

### 2.1 Site-of-Metabolism Prediction

SOM-prediction is formulated as a binary node classification problem on undirected graphs *G* = (*V*, *E*), where each node *v*_*i*_ ∈ *V* represents an atom and each edge *e*_*ij*_ ∈ *E* represents a bond between nodes *v*_*i*_ and *v*_*j*_. Each node carries a binary label *y*_*i*_, where *y*_*i*_ = 1 if the underlying atom is a SOM and 0 otherwise. To address this classification problem within a supervised learning framework, we define a training data set:

$$D≔\{\left(x_{i},y_{i}\right){\}}_{i=1}^{N}\subset X\times Y$$ 1

where X denotes the input space of atom- and bond-level features, and Y represents the outcome space. The training instances (**x**_*i*_,*y*_*i*_) are assumed to be independent and identically distributed according to an unknown probability measure *P* on X×Y , with density function *p*. The objective is to find a function h:X→Y within a hypothesis space H , that maps inputs to outcomes while minimizing a loss function L:Y×Y→R . This objective is formally expressed as

$$h^{*}:=\underset{h\in H}{\mathrm{argmin}}\int_{X\times Y}L\left(h\left(x\right),y\right)dP\left(x,y\right)$$ 2

Since *P* is unknown, training relies on empirical risk minimization:

$$ĥ≔\underset{h\in H}{\mathrm{argmin}}\frac{1}{N}\overset{N}{\underset{i=1}{\sum }}L\left(h\left(x_{i}\right),y_{i}\right)$$ 3

where ĥ represents the empirical risk minimizer, i.e., the best approximation to the true risk minimizer h*, given the available training data D .

### 2.2 Uncertainty Quantification and Decomposition

The following section outlines the different sources of uncertainty in supervised learning, which will be the basis for assessing the uncertainty associated with individual predictions. For a more detailed discussion on how predictive uncertainty can be decomposed into its various sources, we refer the reader to Huellermeier and Waegeman.^32^

#### 2.2.1 Aleatoric Uncertainty

The outcome associated with an instance **x**_*i*_ is typically not a point estimate *y*, but a conditional probability distribution on Y :^32^

$$p\left(y|x_{i}\right)=\frac{p\left(x_{i},y\right)}{p\left(x_{i}\right)}$$ 4

The uncertainty associated with the exact value of *y* is referred to as the aleatoric uncertainty,^31,32,34^ In cases where only point estimates are of interest, predictions are given by the pointwise Bayes predictor f*, which minimizes the expected loss for each x∈X :^32^

$$f^{*}\left(x\right):=\underset{ŷ\in Y}{\mathrm{argmin}}\int_{Y}L\left(ŷ,y\right)dP\left(y|x\right)$$ 5

#### 2.2.2 Model Uncertainty

The uncertainty regarding the gap between the true risk minimizer h* and the pointwise Bayes predictor f* is called model uncertainty or model bias.^32^ It is due to uncertainties in the modeling process itself and reflects our lack of knowledge about the best way to model the underlying task. Factors such as selecting appropriate features and hypothesis space contribute to this type of uncertainty.^34^ In essence, the more domain knowledge the modeler possesses, the lower the model uncertainty is likely to be.

#### 2.2.3 Approximation Uncertainty

The difference between the empirical risk minimizer ĥ and the true risk minimizer h* is called the approximation uncertainty, or model variance.^32,34^ It is heavily influenced by the size of the training data set and is expected to vanish as the amount of training data approaches infinity.^32,34^

Approximation and model uncertainties together form the epistemic uncertainty.^31,32^ The key distinction between aleatoric and epistemic uncertainty is that the latter can, in principle, be reduced with additional information, such as better background knowledge to address model bias or more training data to reduce model variance. In contrast, aleatoric uncertainty is typically considered irreducible, as it stems from the inherent randomness in the data.^31,32,34^

Nevertheless, it is important to recognize that the distinction between aleatoric and epistemic uncertainty is context-dependent and can shift depending on the problem setup.^31,32^ For instance, replacing the instance space X with a more descriptive instance space X′ (e.g., by adding features) can reduce aleatoric or model uncertainty (or both). However, this typically increases approximation uncertainty, as fitting a model with higher-dimensional features becomes more challenging with a fixed amount of data.

Moreover, even within a fixed setup, separating predictive uncertainty into its noise, variance, and bias components is not always straightforward. For example, consider multiple instances with identical features but differing labels. In such cases, it can be challenging to discern whether the uncertainty comes from true randomness (noise) or the modeler’s lack of knowledge about existing but unaccounted differences between these data points (bias). This simple example demonstrates the complications that can arise when attempting to distinguish between the different sources of uncertainty.

### 2.3 Deep Ensembles

Neural networks define a function f:X→Y , parametrized by a set of weights and biases $\omega =\{W_{l},b_{l}{\}}_{l=1}^{L}$ , where *L* denotes the number of layers. Bayesian neural networks (BNNs) treat their parameters as random variables and place a prior distribution *p*(ω) over the network’s parameters^39,40^ unlike conventional neural networks, which treat their parameters as point estimates. Typically, the prior is a zero-mean Gaussian with diagonal covariance, *N* (0, *I*).

According to Bayes’ theorem, the predictive distribution p(y|x,D) for a given input **x** is computed by marginalizing over the posterior distribution p(ω|D) :

$$p\left(y\;|\;x,D\right)=E_{\omega ∼p(\omega |D)}\left[p\left(y\;|\;x,\omega \right)\right]$$ 6

However, this operation is intractable for nearly all neural networks, as it requires computing an integral over the space of all possible model parameters.

Variational inference can be used to approximate the true posterior p(ω|D) with a simpler distribution *q*_0_(ω). This approximation is achieved by indirectly minimizing the Kullback-Leibler (KL) divergence between *q*_0_(ω) and the true posterior, typically through the maximization of the evidence lower bound (ELBO).^41–43^

Sampling methods generate a finite set of network parameters whose empirical distribution approximates the posterior distribution as the number of samples increases. This can be achieved through techniques such as MC-dropout^44,45^ or by ensembling models. Ensembling can involve training with either randomly sampled data (bootstrapping) or the same data but different initial random weights (deep ensembling).^38^ Although originally proposed as a non-Bayesian method, ensembling has been shown to offer similar advantages to Bayesian inference, and to provide more reliable uncertainty estimates than MC-dropout in practice.^46^

Kendall and Gal^47^ demonstrated how to model both epistemic and aleatoric uncertainties in deep learning using MC-dropout and maximum a posteriori (MAP) estimation inference, respectively. In particular, the authors advocated for a distinction between aleatoric and epistemic uncertainty when using deep-learning methods to remedy neural networks’ inability to recognize and quantify their own uncertainty. Zhang and Lee^33^ and Ryu et al.^37^ independently adapted this strategy to predict continuous-valued molecular properties with uncertainty quantification using graph neural networks. Scalia et al.^27^ further refined the approach, demonstrating that the ensemble-based approach proposed by Lakshminarayanan et al.^38^ outperformed MC-dropout for deep learning-based molecular property regression.

Deep ensembles model epistemic uncertainty by analyzing the behavior of individual models trained on the same data but with different random initializations. When all models agree on their predictions, it suggests a clear and well-understood relationship between the input features and the labels, indicating low uncertainty. On the other hand, if the models produce divergent predictions, it signals that they may struggle to learn a robust representation for the data, which could be due to insufficient similar training data or inadequately descriptive input features.

In classification settings, the total predictive uncertainty is often measured using the entropy over the predictive distribution p(y|x,D) :^48^

$$H\left[p\left(y\;|\;x,D\right)\right]=-\underset{y\in Y}{\sum }p\left(y\;|\;x,D\right)\log p\left(y\;|\;x,D\right)$$ 7

Moreover, mutual information (MI) can be used to separate the variance-related from the nonvariance-related contributions to the total uncertainty.^48–50^ The MI between the model parameters ω and a label *y* for some new data point **x** given the data set D is

$$I\left(\omega ,y\;|\;x,D\right)=H\left[p\left(y\;|\;x,D\right)\right]-E_{p(\omega \;|\;D)}\left[H\left[p\left(y\;|\;x,\omega \right)\right]\right]$$ 8

Here, the predictive entropy H[p(y|x,D)] represents the total uncertainty, the expected value of the entropy $E_{p(\omega |D)}\left[H\left[p\left(y\;|\;x,\omega \right)\right]\right]$ represents the nonvariance-related, and the MI the variance-related part of the total predictive uncertainty.^48–50^

The variance-related part of the uncertainty is equivalent to the approximation uncertainty, while the nonvariance-related part encompasses both the aleatoric and model uncertainties.^32,34^ In practice, it is often assumed that the hypothesis space H is correctly specified, meaning $f^{*}\in H$ . This is especially plausible in deep learning, where models are generally believed to have ″universal approximation″ capabilities.^32^ This assumption makes it possible to ignore model uncertainty, which is very difficult to capture.^32^ Thus, variance- and nonvariance-related uncertainties are referred to as epistemic and aleatoric uncertainties, respectively.

These quantities, analytically intractable for deep networks, are approximated via MC-dropout or deep ensembles. Specifically, the predictive distribution p(y|x,D) is approximated by computing the Bayesian model average (BMA) of the predictions $p\left(y\;|\;x,\omega_{m}\right)$ :

$$p\left(y\;|\;x,D\right)≃\frac{1}{M}\overset{M}{\underset{m=1}{\sum }}p\left(y\;|\;x,\omega_{m}\right)$$ 9

where *M* and ω_*m*_ are the number of individual models in the ensemble and the parameters of the *m* ^*th*^ model, respectively.

The total uncertainty *u*_*tot*_ is obtained by computing the entropy of the BMA:

$$u_{\mathrm{tot}}=H\left[\frac{1}{M}\overset{M}{\underset{m=1}{\sum }}p\left(y\;|\;x,\omega_{m}\right)\right]$$ 10

Analogously, the aleatoric uncertainty *u*_*ale*_ is the average of the entropy of the individual predicted distributions:

$$u_{\mathrm{ale}}=\frac{1}{M}\overset{M}{\underset{m=1}{\sum }}H\left[p\left(y\;|\;x,\omega_{m}\right)\right]$$ 11

Finally, the epistemic uncertainty *u*_*epi*_ is the difference between total and aleatoric uncertainty:

12 $$u_{\mathrm{epi}}=u_{\mathrm{tot}}-u_{\mathrm{ale}}$$

A schematic depiction of the modeling of aleatoric and epistemic uncertainty in aweSOM is given in Figure 1.

**Figure 1:**
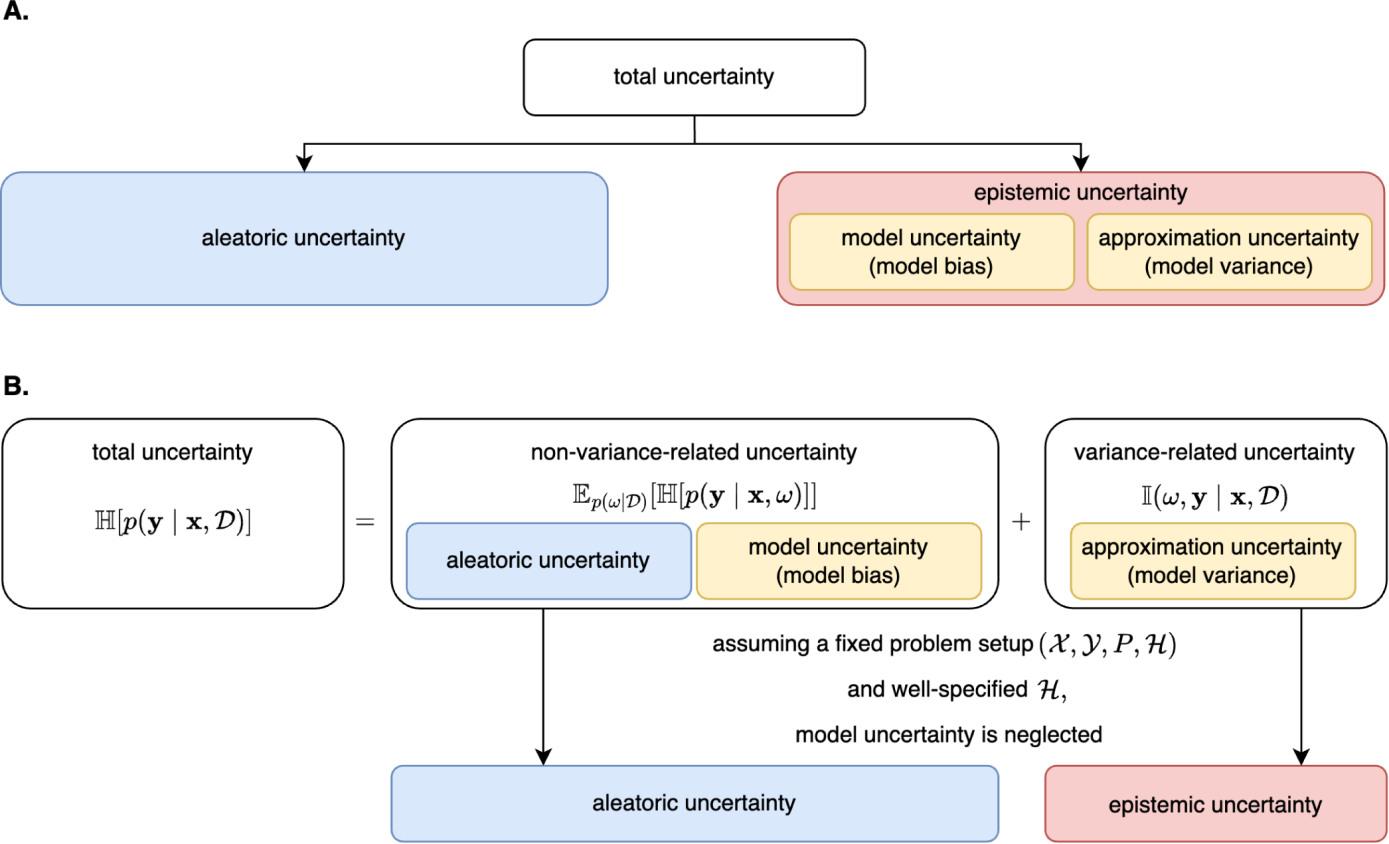
Schematic representation of aleatoric and epistemic uncertainty modeling in aweSOM. The data set $D≔{\left\{\left(x_{i},y_{i}\right)\right\}}_{i=1}^{N}\subset X\times Y$ consists of input instances x⊂X and corresponding outcomes y⊂Y . The data points are assumed to be independently and identically distributed according to an unknown probability measure *P* over X×Y , with density function *p*. The model parameters are denoted by *ω*, H ­[·] represents entropy, E ­[·] the expected value, and I ­[·] the mutual information. **A.** Total predictive uncertainty is decomposed into aleatoric and epistemic components. **B.** Deep ensembling is employed to model both aleatoric and epistemic uncertainties in aweSOM.

### 2.4 Data

To evaluate our approach, we employed the MetaQSAR database.^51,52^ The current version comprises 2,825 parent compounds annotated with 6,320 expert-curated SOMs related to mammalian phase I and phase II metabolism observed *in vivo* or *in vitro*. After preprocessing (procedure described in Section S2), the MetaQSAR-derived training set contains 1,971 compounds, and the test set 360 compounds. The compounds have, on average, 2.4 SOMs. Among heavy atoms, the ratio of SOMs to non-SOMs is approximately 1:10. Given the lack of data on metabolically stable compounds, the data set does not include nonmetabolized compounds.

Moreover, we should note that the MetaQSAR database has been expanded and revised since it was used for the development and validation of FAME3 ^25^ and FAME.AL.^53^ To support the comparability of performance tests reported in this work and the previous studies, any new data was added to the training set while the test set described in ref 25 and ref 53 was preserved.

### 2.5 Model Architecture and Training

aweSOM is an ensemble of 10 GNN-models, each trained with different random initializations. Each model consists of three core modules: a convolutional module, a molecular context pooling module, and a classification module. A schematic depiction of the model architecture is presented in Figure 2.

**Figure 2:**
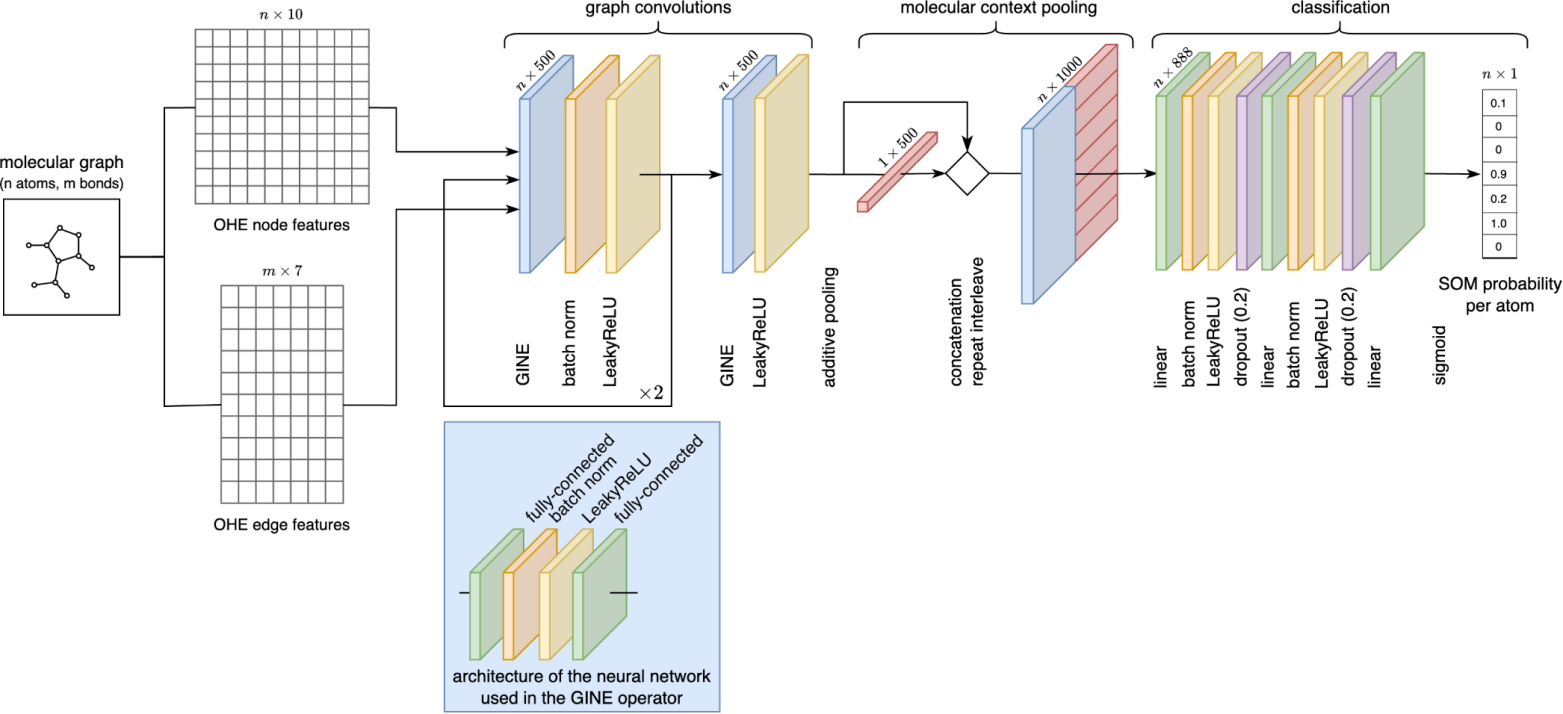
Schematic representation of aweSOM’s architecture.

The convolutional module includes four GINE layers of size 500, interspersed with batch normalization and LeakyReLU activation.^54^ The molecular context-pooling module generates a graph-level representation via additive pooling. This pooled representation is concatenated to all node-level representations in the graph, effectively doubling their dimensionality. The classification module consists of two fully connected layers with 888 neurons, interspersed by batch normalization, LeakyReLU activation, and dropout layers, followed by a final fully connected layer that maps the latent representation to a probabilistic output using the sigmoid function. Detailed information on hyperparameter optimization is provided in Section S3.

The training was conducted using the AdamW optimizer^55^ a weighted binary cross-entropy loss (positive class weight: 2.1) and a batch size of 32. The weighting of the loss addresses the class imbalance, allowing a binary classification threshold of 0.5 for predictions. The learning rate and number of epochs were determined during the hyperparameter optimization phase via 10-fold cross-validation, details of which can be found in Section S3.

### 2.6 Benchmarking and Evaluation Metrics

FAME3R is a random forest classifier with 250 estimators. The input data is featurized using FAME fingerprints (circular, atom-based binary fingerprint with a radius of 5) and 14 electronic and topological descriptors. To address the class imbalance (1:10), two strategies are employed: during training, it assigns weights inversely proportional to class frequencies within each bootstrap sample for every tree, and during postprocessing, it applies a binary decision threshold of 0.3. All of this approach and setup is consistent with that of FAME3, for which full detail is provided in Šícho et al.^25^

FAME3R’s and aweSOM’s predictive performance was assessed using the area under the receiver operating characteristics curve (ROC-AUC), area under the precision-recall curve (PR-AUC), F1-score, Matthews correlation coefficient (MCC), precision, recall, and top-2 success rate (TOP-2). The MCC, ranging from −1 to +1, is a comprehensive measure for evaluating binary classifiers, particularly on unbalanced data sets. Recall measures the proportion of positive samples recovered by the model, precision quantifies the proportion of true positives among all predicted positives. The ROC-AUC reflects the model’s ability to rank positive samples above negative ones. The top-2 success rate captures the proportion of molecules for which at least one known SOM is included among the two top-ranked atom positions.

## 3 Results

### 3.1 Benchmarking aweSOM against FAME3R

We benchmarked the performance of aweSOM against FAME3R (see Section 2.6 for details). As summarized in Table 1, aweSOM achieved classification and ranking performances comparable to FAME3R, with nearly identical metrics and overlapping standard deviations on the test and validation sets. Specifically, the MCCs on the test for aweSOM and FAME3R were 0.52 ± 0.01 and 0.48 ± 0.01, respectively. Likewise, the PR-AUC values were 0.56 ± 0.02 for aweSOM and 0.57 ± 0.02 for FAME3R. The top-2 success rates were 0.82 ± 0.02 for both models, indicating that for over 80% of test compounds, at least one known SOM was correctly identified among the top-2 atom positions. Taking into account the challenges in fully annotating SOMs due to experimental limitations, these results likely provide a conservative estimate of model performance.

**Table 1:** Benchmarking aweSOM against FAME3R

	aweSOM		FAME3R	
	10-fold CV^a^	test^b^	10-fold CV^a^	test^b^
ROC-AUC	0.88 ± 0.04	0.89 ± 0.01	0.88 ± 0.01	0.89 ± 0.01
PR-AUC	0.53 ± 0.08	0.56 ± 0.02	0.53 ± 0.03	0.57 ± 0.02
F1	0.52 ± 0.06	0.58 ± 0.01	0.53 ± 0.02	0.54 ± 0.01
MCC	0.46 ± 0.07	0.52 ± 0.01	0.48 ± 0.02	0.48 ± 0.01
Precision	0.51 ± 0.09	0.55 ± 0.01	0.47 ± 0.05	0.57 ± 0.01
Recall	0.53 ± 0.05	0.60 ± 0.02	0.62 ± 0.06	0.63 ± 0.02
TOP-2	0.72 ± 0.09	0.82 ± 0.02	0.80 ± 0.02	0.82 ± 0.02

a Mean and standard deviation were calculated from the 10 cross-validation folds.

b Mean and standard deviation were calculated from 1,000 bootstrap samples, each drawn with replacement from the test set, containing 360 molecules.

From a technical perspective, the key difference between the two models lies in their classification strategies. aweSOM employs a multilayer perceptron (MLP) module that classifies end-to-end learned atomic representations, capturing both atom type and structural environment within a radius determined by its convolutional layers. In contrast, FAME3R uses a random forest to classify atom-centered fingerprints encoding similar structural information within a predefined radius. The comparable performance of these approaches is not surprising, as no theoretical basis suggests a significant divergence, despite contrary claims.^24^ The primary advantage of aweSOM’s architecture is its robust uncertainty quantification.

### 3.2 Correlation between Uncertainty and Predictive Accuracy

It is reasonable to expect predictions with lower uncertainty to be more likely accurate. To investigate whether a negative correlation exists between uncertainty and aweSOM’s predictive performance, we sorted the 8,095 atoms in the test set based on their uncertainty scores and computed the Brier score for increasing percentages of the total test set. The Brier score is a strictly proper scoring rule that measures the accuracy of probabilistic predictions. For unidimensional predictions, it is mathematically equivalent to the mean squared error applied to predicted probabilities.

As shown in Figure 3, when considering only the most certain data points, the Brier score is close to zero, indicating near-perfect predictions. However, as more data points are included (moving toward the upper-right point, which represents the entire test set) the Brier score increases, signifying that aweSOM made more incorrect predictions. The curves in Figure 3 exhibit a predominantly increasing trend, confirming that higher uncertainty is associated with lower predictive accuracy. In Section 3.5, we provide practical guidance on how to interpret and act on different levels of epistemic and aleatoric uncertainty based on empirical thresholds derived from our data set.

**Figure 3:**
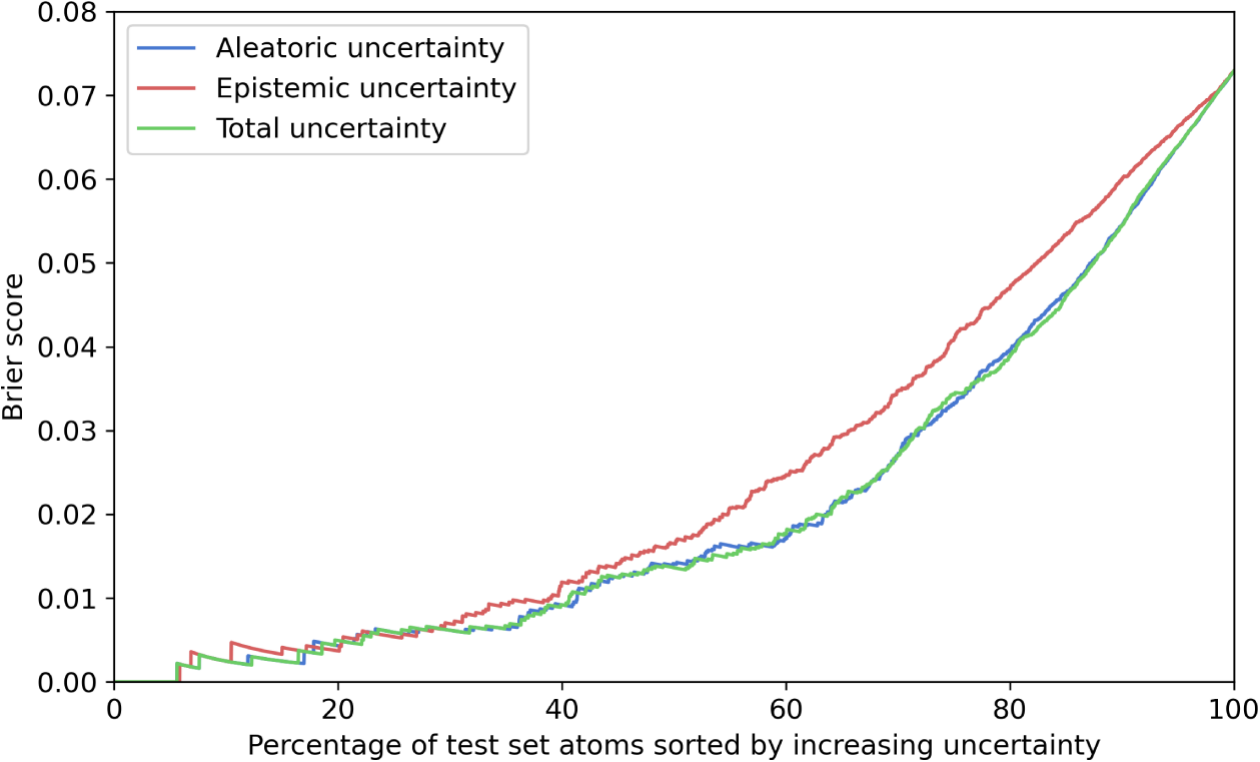
Brier score as a function of the percentage of the test set sorted by uncertainty. Lower uncertainty is associated with higher predictive accuracy, as evidenced by the predominantly increasing trend of the curves. This holds true for the aleatoric, epistemic, and total uncertainty.

### 3.3 Controlled Studies for Understanding Epistemic and Aleatoric Uncertainties

Since our data lacks uncertainty estimates, we conducted two experiments using synthetic data to validate our uncertainty decomposition strategy. The first experiment examined the effect of data coverage on epistemic uncertainty, which typically increases when relevant training data is missing. To test this, we trained a ″halogen-agnostic″ model on a subset of the training data that excluded all molecules containing halogen atoms (F, Cl, Br, and I). The second experiment focused on aleatoric uncertainty by introducing artificial label noise. We created a ″noise-injected″ model by modifying the training set: all substrates containing at least one halogen atom were duplicated, with the duplicates intentionally mislabeled as having halogen atoms as SOMs. As a result, the training set contained an equal number of halogen atoms labeled as SOMs and non-SOMs.

Halogen atoms were selected for these experiments for two reasons. First, they are consistently labeled as non-SOM in the original data set, making it easier to control the level of artificial noise. Second, they appear in approximately 30% of all substrates, allowing us to exclude or relabel a significant subset of molecules without compromising model training. This setup provides a robust framework for demonstrating how different types of uncertainty respond to missing or noisy data in metabolism prediction.

Following hyperparameter tuning and model training (see Section S3 for details), we evaluated the halogen-agnostic and noise-injected models using the original test set, where halogen-related modifications were not applied. As shown in Table 2, performance metrics declined slightly in both experiments. For instance, the MCCs dropped from 0.52 to 0.45. The drop might appear less dramatic than expected, given the amount of tempered substrates. However, halogen atoms represent less than 3% of the atoms in the test set (221 out of 8,095), posing challenges to the detection of differences using standard metrics alone.

**Table 2:** Performance on the Test Set of the Halogen-Agnostic and Noise-Injected Models Compared to aweSOM (Baseline)^a^

	baseline	halogen-agnostic	noise-injected
ROC-AUC	0.89 ± 0.01	0.86 ± 0.01	0.87 ± 0.01
PR-AUC	0.56 ± 0.02	0.51 ± 0.02	0.54 ± 0.02
F1	0.58 ± 0.01	0.51 ± 0.01	0.51 ± 0.01
MCC	0.52 ± 0.01	0.45 ± 0.02	0.45 ± 0.02
precision	0.55 ± 0.01	0.53 ± 0.02	0.46 ± 0.01
recall	0.60 ± 0.02	0.49 ± 0.02	0.58 ± 0.02
TOP-2	0.82 ± 0.02	0.77 ± 0.02	0.80 ± 0.02
${\mathrm{p̅}}_{\mathrm{hal}}$ ^b^	0.01 ± 0.03	0.35 ± 0.23	0.70 ± 0.03
$N_{\mathrm{hal}}^{\mathrm{pos}}$ ^c^	0	50	216
$N_{\mathrm{hal}}^{\mathrm{neg}}$ ^d^	216	166	0

a All models were built using the GINE convolutional operator with context pooling.

b Average predicted SOM-probability of all halogen atoms.

c Number of predicted positive halogens.

d Number of predicted negative halogens.

To gain more insights into the impact of the modified training data on model performance, we analyzed the average predicted SOM-probabilities for halogen atoms and examined the distribution of halogens classified as SOMs or non-SOMs. The results show clear patterns: the model trained on unmodified data correctly classified all halogen atoms as negatives. In contrast, the halogen-agnostic model produced almost random predictions, with an average SOM-probability of 0.35. Meanwhile, the noise-injected model classified all halogen atoms as being positive. Boxplots of the predicted SOM-probabilities per atom type for aweSOM, the halogen-agnostic and the noise-injected models can be found in Section S4.

This analysis highlights the significant impact of data quality on model performance, as well as the limitations of traditional performance metrics at relating poor predictive outcomes to underlying issues with insufficient data coverage or noise. This underscores the need for comprehensive uncertainty measures, which are crucial for diagnosing model failures and gaining deeper insights into model behavior.

Figure 4 presents the evolution of aleatoric and epistemic uncertainty across the three experimental scenarios (baseline, halogen-agnostic, and noise-injected). All panels show the uncertainty distributions by atom type (boxplots), which include the halogens (fluorine, chlorine, bromine, iodine), as well as carbon, nitrogen, oxygen, and sulfur, categorized by hybridization state (e.g., ″C-SP″ for sp-hybridized carbon).

**Figure 4:**
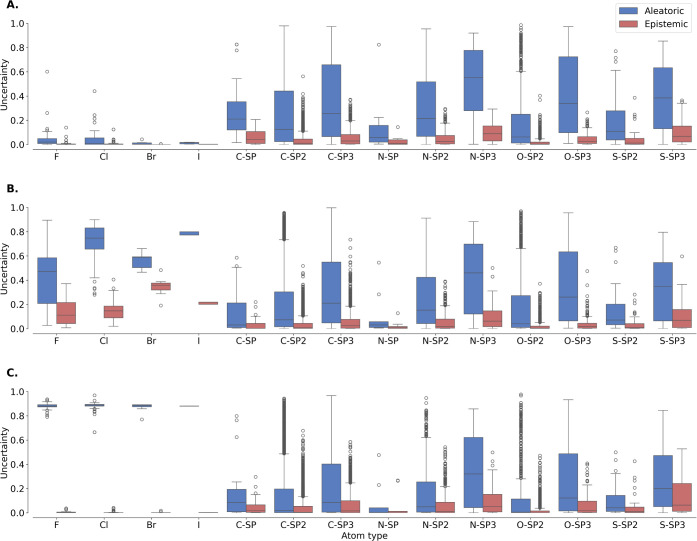
Comparison of aleatoric and epistemic uncertainties by atom type across different experimental scenarios. **A**. Baseline scenario serving as the reference point for our study of uncertainties. **B**. Halogen-agnostic scenario, where molecules containing halogen atoms are removed from the training set while the test data remains untouched. This setup evaluates the impact of missing halogen-containing examples on model uncertainties, focusing on epistemic uncertainty. The results show increased epistemic uncertainty for halogen atoms, reflecting the model’s reduced confidence and increased uncertainty due to the absence of relevant training examples. **C**. Noise-injected scenario, where the training data set includes halogen atoms with artificially increased label noise, with the test data remaining untouched. This scenario examines how label noise affects uncertainties, focusing on aleatoric uncertainty. The results indicate a notable increase in aleatoric uncertainty for halogen atoms, highlighting the sensitivity of the model to label noise and its effect on predictive variability.

As hypothesized, injecting noise into the SOM labels (Figure 4, panel C) significantly increased aleatoric uncertainty without affecting epistemic uncertainty. Specifically, the mean aleatoric uncertainty for halogen atoms collectively jumped from 0.04 ± 0.07 to 0.88 ± 0.03, while epistemic uncertainty decreased from 0.01 ± 0.02 to 0.004 ± 0.007. In the halogen-agnostic scenario (Figure 4, panel B), the absence of relevant training data led to an increase in epistemic uncertainty, and a less substantial increase in aleatoric uncertainty, compared to the noise-injected scenario. Here, mean aleatoric uncertainty for halogen atoms rose from 0.04 ± 0.07 to 0.6 ± 0.2, while mean epistemic uncertainty increased from 0.01 ± 0.02 to 0.2 ± 0.1. Both uncertainty types remained stable for other atom types, indicating that these changes were specific to halogen atoms.

One of the above findings was rather unexpected, as we initially hypothesized that the halogen-agnostic scenario would yield epistemic uncertainties close to one and aleatoric uncertainties close to zero for halogen atoms. However, the observed epistemic uncertainty values averaged 0.2, while aleatoric uncertainty values averaged 0.6. In the following, we propose three possible explanations.

The first explanation is based on a mathematical argument. Given that epistemic uncertainty is computed as the difference between the Shannon entropy of the BMA and the expected Shannon entropy across individual model predictions, for it to reach one, exactly half of the models in the ensemble must output a SOM probability of zero, while the other half must output one, which is a highly improbable scenario given that the weights associated with missing instances (in our case halogen atoms) cannot be trained. Instead, the individual models in the ensemble output random values when confronted with halogen atoms at inference time. This hypothesis is consistent with the discussion proposed by Yang and Li in their work on uncertainty quantification in deep learning-based molecular property regression tasks.^35^ If we assume that the corresponding logits are approximately uniformly distributed between *x* and -*x* (for some *x* ∈ R ), which is plausible given the variability in neural network outputs for out-of-distribution data, we can derive theoretical maximum values for epistemic uncertainty. For example, if *x* = 1, the maximum value is 0.06; if *x* = 5, it is 0.54; and if *x* = 10, it is 0.76.

The second explanation relates to the nature of preclassification embeddings, which are influenced not only by the atoms themselves but also by their surrounding chemical environment. The size of this neighborhood is determined by the number of convolutional layers (in our case, four layers). Consequently, even though all molecules containing halogen atoms were removed from the training data, the embeddings of halogen atoms in the test set were not entirely novel to the model. The presence of partially learned molecular fragments in the halogens’ neighborhood reduced the likelihood of maximum epistemic uncertainty.

The third explanation pertains to the underlying assumptions that the hypothesis space H is well-defined and that the problem setup ( X , Y , P , H ) is fixed. These assumptions allowed us to equate aleatoric and epistemic uncertainty with the nonvariance and variance components of total predictive uncertainty, respectively. However, if the latter assumption is relaxed, nonvariance-related uncertainty encompasses both model uncertainty and aleatoric uncertainty (see Figure 1 and Section 2.3). Since we modified the problem setup by altering the training data (dropping almost 30% of it), the assumption that model uncertainty is negligible no longer holds. Consequently, the observed increase in epistemic uncertainty may reflect a stronger contribution from model uncertainty rather than approximation uncertainty. This interpretation aligns with findings by Heid et al.^34^ who demonstrated that in small data sets, epistemic uncertainty is predominantly driven by model uncertainty.

Overall, these experiments demonstrate that missing relevant training data increases epistemic uncertainty, whereas label noise primarily affects aleatoric uncertainty. These findings highlight the importance of comprehensive uncertainty estimation in model evaluation, extending beyond traditional performance metrics.

### 3.4 Uncertainty Decomposition: Identifying Error Sources to Guide Model Improvements

Building on the insights from the halogen experiments, we analyzed the distribution of aleatoric and epistemic uncertainties in aweSOM to identify whether errors are driven by limitations in the training set or inherent noise in the data. Identifying the dominant type of uncertainty is valuable, as it reveals the root causes of errors and informs strategies for model improvement.

The results are shown in Figure 5. Panel A presents the correlation between uncertainty type and predicted SOM probabilities, while panel B displays the uncertainties’ probability density functions. These plots suggest that aleatoric uncertainty is the predominant source of uncertainty in SOM predictions, which aligns with the findings of Scalia et al.^27^ who evaluated various uncertainty estimation methods for deep learning-based molecular property prediction. The aforementioned study demonstrated that the relative contributions of aleatoric and epistemic uncertainty depend on the data set, with epistemic uncertainty being more pronounced in computationally derived data (e.g., quantum mechanical properties) and aleatoric uncertainty prevailing in experimental data sets. Our results support this trend, indicating that in our setting, where labels are experimentally derived, aleatoric uncertainty is the primary contributor to total uncertainty.

**Figure 5:**
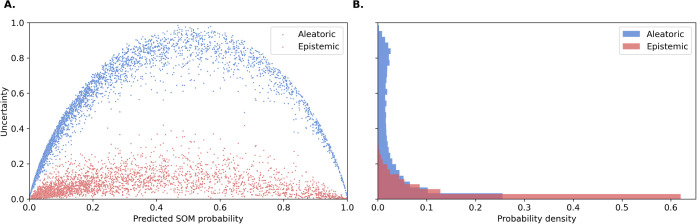
Distribution of aleatoric and epistemic uncertainties across atoms in the test set.

Deep ensembling is a well-established approach for reducing epistemic uncertainty.^34^ Ensembling mitigates uncorrelated errors between individual models, leading to performance improvements when model variance is the primary driver of uncertainty. The results in Table 3 show that the MCC improves as the ensemble size increases from 1 to 10 models, suggesting that model variance contributes to uncertainty in aweSOM. Consequently, expanding the training data set to include underrepresented substructures and metabolic reactions is likely to enhance predictive performance.

**Table 3:** Matthews Correlation Coefficient versus Ensemble Size

Ensemble size	1	5	10	50	100
MCC	0.47 ± 0.01	0.50 ± 0.01	0.52 ± 0.01	0.52 ± 0.01	0.52 ± 0.01

To simulate the effect of increasing training data, we trained models on subsets of the available data set, ranging from 10% to 100% of the full training set. As shown in Figure 6, test set performance generally improves with increasing training data. However, performance begins to plateau at higher data set sizes, suggesting diminishing returns. For additional training data to be beneficial, it should specifically target substrates with rare substructures, which could be identified, for example, by selecting compounds structurally similar to those containing atoms with high epistemic uncertainty.

**Figure 6:**
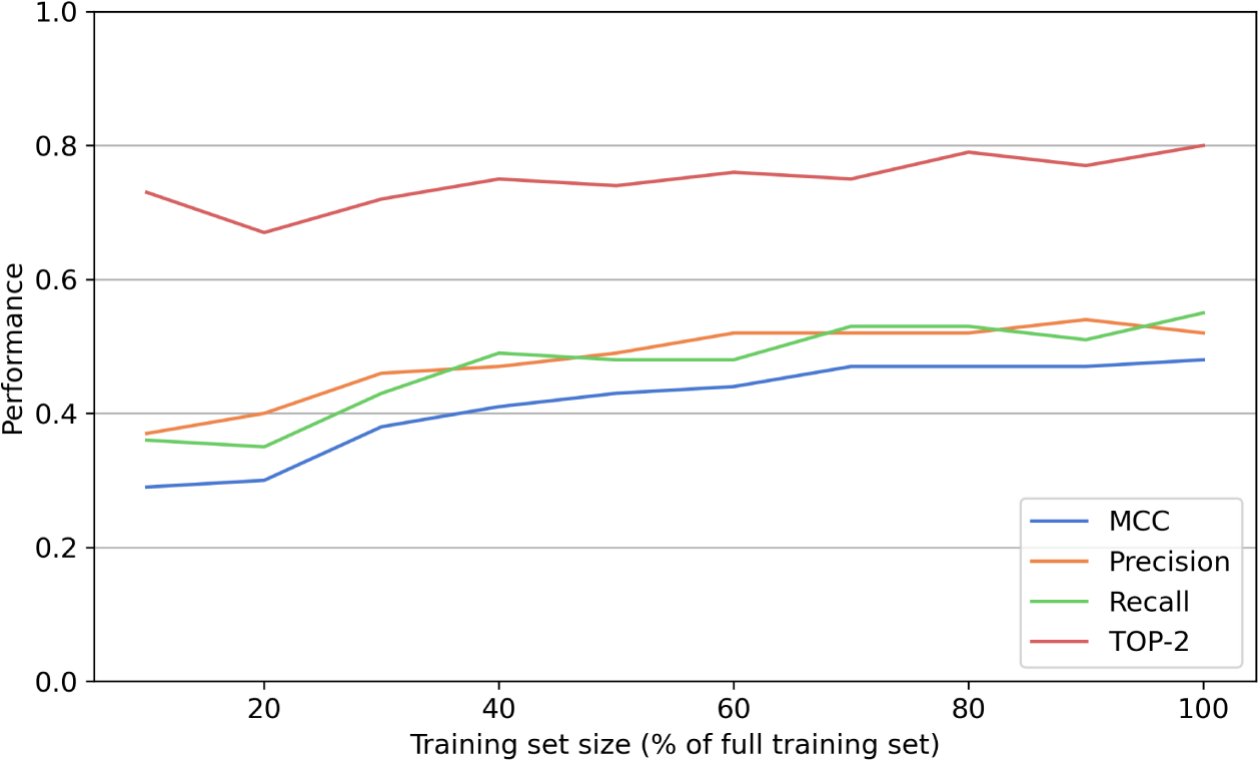
Effect of training data size on model performance. This plot shows aweSOM’s performance on the test set as a function of the proportion of randomly sampled data from the training set. Standard deviations were omitted as none exceeded 0.02.

### 3.5 Case Studies

We illustrate the behavior of aweSOM and its capabilities for UQ at the example of three molecules (Figure 7): abemaciclib, a cyclin-dependent kinase (CDK) inhibitor for the treatment of metastatic breast cancers; etrasimod, a sphingosine 1-phosphate (S1P) receptor modulator used to treat ulcerative colitis; and 2,4,6-trichlorobiphenyl (PCB 30), a compound formerly used in industrial and consumer products.

**Figure 7:**
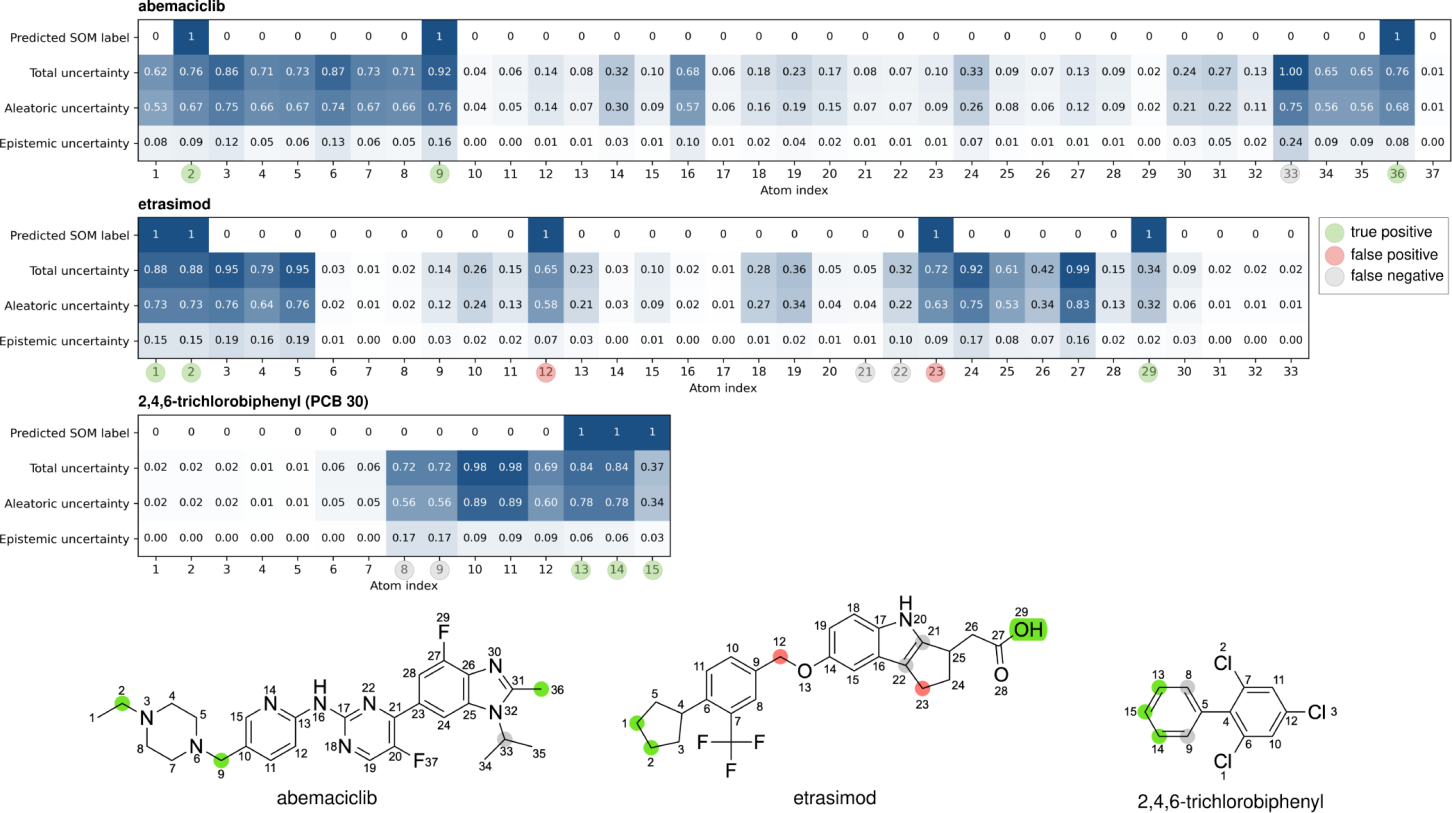
Analysis of model predictions and uncertainty scores for three xenobiotics: abemaciclib, etrasimod, and PCB 30.

We present these particular molecules to highlight how aleatoric and epistemic uncertainties scale differently, emphasizing the importance of evaluating them separately. In the MetaQSAR test set, the epistemic uncertainty values ranged from 0 to 0.56, with a 95th percentile value of 0.16, while aleatoric uncertainty values ranged from 0 to 0.97, with a 95th percentile value of 0.85. Our recommendation for interpreting this information is first to evaluate epistemic uncertainty to determine whether it falls within an acceptable range, as it reflects the adequacy of training data coverage. While the definition of an acceptable range may vary based on user-specific criteria, we consider values below 0.10 to indicate sufficient representation in the training data. Only if epistemic uncertainty is low, suggesting that the model has been exposed to a sufficient number of similar instances, should aleatoric uncertainty be evaluated. In this context, we consider aleatoric uncertainty values above 0.70 to be indicative of unreliable predictions.

According to Thakkar and Kate^56^ the metabolism of abemaciclib in rats and human liver microsomes produces up to 11 first-generation metabolites. Two of these metabolites have unresolved structures, and five result from combinations of reactions, leaving four distinct reactions (and thus four SOMs) in total. In human liver microsomes, the major metabolites are N-desethylabemaciclib and hydroxyabemaciclib, the latter arising from the hydroxylation of the methyl group on the benzimidazole moiety. As shown in Figure 7, aweSOM correctly predicted both SOMs associated with these two metabolites. Additionally, it predicted the SOM for a minor metabolite formed through oxidative deamination of the piperazine moiety. However, it failed to identify the SOM for the N-dealkylation of the benzimidazole moiety.

Analyzing the corresponding uncertainties reveals that the missed SOM has a total uncertainty score of 1.00, indicating a highly unreliable prediction. Decomposing the uncertainty scores further, we observe that the epistemic uncertainty is high (0.24), suggesting that aweSOM lacked sufficient similar examples in the training data to make an informed prediction for this atom. For users who can generate training data, this highlights the need to test molecules with similar structural patterns and incorporate the results into future model iterations to enhance performance.

The predictions for abemaciclib demonstrate that positive predictions are often associated with high aleatoric and total uncertainty scores. This should be expected, as uncertainty scores are calibrated across all atoms in the training set, the majority of which are trivial to predict, such as halogens or quaternary carbon atoms that are never metabolized, thus receiving the lowest uncertainty scores. At the same time, when an atom exhibits some degree of reactivity, model uncertainty increases accordingly. Therefore, aleatoric uncertainty values in the range of 0.5–0.6 should not be considered particularly concerning, as they reflect expected variability rather than a fundamental limitation of the model.

PCB 30 showcases how aleatoric uncertainty scores can be used to identify unreliable predictions. In this case, aweSOM correctly predicted three of the five experimentally reported SOMs but failed to capture two at the ortho-positions of the nonsubstituted phenyl ring. Additionally, it nearly produced two false positives at the meta-positions of the substituted phenyl ring, as indicated by the high total uncertainty scores (0.98).

The elevated aleatoric uncertainty scores for atoms 10, 11, 13, and 14 signal significant variability in SOM annotations across the 21 polychlorinated biphenyls (PCBs) in the training data. Notably, seven of these training PCBs contain a nonsubstituted phenyl ring, like PCB 30, yet exhibit markedly different oxidation patterns. Similarly, 12 training PCBs feature a trichlorophenyl moiety with oxidation potential at the meta position, but such reactions are only documented in eight cases. The only reliable predictions in this case are the absence of reactions at atoms 1–7 and 12, as well as the reaction at atom 15.

PCB 30 is a clear example of how SOM annotation is influenced not only by molecular reactivity but also by limitations in experimental data collection, interpretation, and reporting. In some cases, particularly for oxidation reactions on aromatic rings, precise SOM determination may not be feasible from available experimental data. Ambiguity in such cases often leads to inconsistent annotation strategies, where experts may either annotate all plausible positions, rely on their best judgment, or omit SOMs altogether. These inconsistencies can significantly impair model performance, especially in low-data regimes. This case study underscores the importance of consistent and rigorous data curation in improving the reliability of metabolism predictions.

Finally, etrasimod provides an instance of how aweSOM can assist scientists in elucidating the structures of the metabolites of new compounds. A recent study of etrasimod’s metabolism in humans^57^ detected up to 31 metabolites in plasma, feces, and urine, though only 14 were ″definitively or tentatively″ identified. The reported metabolic pathways confirm that three of the SOMs predicted by our model (atoms 1, 2, and 29) correspond to experimentally confirmed SOMs. However, aweSOM also produced one false positive (atom 12) and two false negatives (atoms 21 and 22). More importantly, this example highlights how false positives with moderate total uncertainty scores can be used to find sites where experimental methods may have been unable to pinpoint the exact structures of the metabolites, even though reactions were detected. Specifically, atom 23, which was predicted to be a SOM with a total uncertainty of 0.72, could correspond to the oxidation site in the unresolved oxidation reaction of the cyclopentindole moiety. This analysis demonstrates how aweSOM can support experimentalists by guiding targeted follow-up studies on specific atoms, ultimately facilitating the comprehensive structural elucidation of all relevant metabolites.

## 4 Conclusions

This work introduces aweSOM, a GNN-based model for predicting phase I and phase II SOMs in xenobiotics with integrated uncertainty estimation. Unlike previous approaches, aweSOM provides comprehensive predictions of potential SOMs across a wide range of xenobiotic-metabolizing enzymes while generating atom-based uncertainty scores. Specifically, aweSOM leverages deep ensembling to model predictive uncertainty and partition it into its aleatoric and epistemic components. This decomposition is used to identify the primary sources of uncertainty for individual predictions.

We show that high epistemic uncertainty is indicative of insufficient training data, while high aleatoric uncertainty is associated with noisy data. Aleatoric uncertainty is generally defined as irreducible noise inherent in the data. However, in the context of SOM prediction, this raises the question of what constitutes “inherent″ noise. In our modeling approach, aleatoric uncertainty reflects a combination of biological variability, limitations in experimental precision, and systematic inconsistencies in data collection and curation. While this interpretation is broader than the conventional definition, it enables us to identify and address sources of variability that affect prediction reliability. When aleatoric uncertainty is high, standardizing SOM annotations across data sources and conducting repeated experiments can help reduce experimental variability and improve labeling consistency. To reduce epistemic uncertainty, expanding training data with rare substructures or uncommon biotransformations is essential. Given the high data acquisition costs, prioritizing regions with high epistemic uncertainty should maximize information gain and ensure efficient use of resources.

To support the practical application of our method, we propose the following interpretation framework: begin by assessing the epistemic uncertainty score. Values below 0.10 can be considered indicative of sufficient data coverage. If the epistemic uncertainty is low, proceed to evaluate the aleatoric uncertainty score. In this case, values below 0.70 can be interpreted as indicative of reliable predictions. Note that these thresholds are based on the results presented in this work and should be adjusted to align with the specific requirements of each user.

In summary, this work introduces aweSOM, a SOM prediction model that decomposes predictive uncertainty into its aleatoric and epistemic components, thereby providing valuable insights into the reliability of individual predictions and highlighting key areas for improvement in data quality and model training. Our findings emphasize the importance of addressing aleatoric uncertainty through improved data curation and standardization, while also reducing epistemic uncertainty by expanding training data sets to include underrepresented chemical spaces and reaction types. Ultimately, we anticipate that aweSOM and the insights presented in this work will contribute to advancing the field of metabolism prediction, enabling more accurate and reliable predictions for a diverse range of xenobiotics.

## Supplementary Material



## Data Availability

The source code of the approach presented in this work is available at https://github.com/molinfo-vienna/aweSOM. The repository also provides access to aweSOM models trained on the publicly available SOM data sets reported in ref 13. The use of aweSOM trained with MetaQSAR data requires a license for MetaQSAR.

## Notes

Further details on software used in this work, data preprocessing, data set statistics, network architecture, performance metrics, and complementary information on aweSOM’s predictions (PDF)
