# Supplementary material for: Site-of-Metabolism Prediction with Aleatoric and Epistemic Uncertainty Quantification
Source: J Chem Inf Model. 2025 Aug 6;65(16):8462–74. doi: 10.1021/acs.jcim.5c00762 (PMC12381852; doi:10.1021/acs.jcim.5c00762)
Supplement: Supplementary file 1 [file ci5c00762_si_001.pdf]

Site-of-Metabolism Prediction with Aleatoric and Epistemic Uncertainty Quantification

Roxane Axel Jacob,[†,‡,⊥] Oliver Wieder,[†,‡] Ya Chen,[†] Angelica Mazzolari,[¶] Andreas Bergner,[§] Klaus-Juergen Schleifer,[‖] and Johannes Kirchmair[*,†,‡]

†*Department of Pharmaceutical Sciences, Division of Pharmaceutical Chemistry, Faculty of Life Sciences, University of Vienna, Josef-Holaubek-Platz 2, 1090 Vienna, Austria*

‡*Christian Doppler Laboratory for Molecular Informatics in the Biosciences, Department of Pharmaceutical Sciences, University of Vienna, Josef-Holaubek-Platz 2, 1090 Vienna, Austria*

¶*Dipartimento di Scienze Farmaceutiche, Universita degli Studi di Milano, I-20133 Milano, Italy*

§*Drug Discovery Sciences, Boehringer Ingelheim RCV GmbH & Co KG, Vienna, Austria*

‖*BASF SE, 67063 Ludwigshafen am Rhein, Germany*

⊥*Vienna Doctoral School of Pharmaceutical, Nutritional and Sport Sciences, University of Vienna, Josef-Holaubek-Platz 2, 1090 Vienna, Austria*

E-mail: johannes.kirchmair@univie.ac.at

S1 Software

Data preprocessing was performed in Python (v.3.10.6) using RDKit (v.2023.3.3) and scikit learn (v.1.6.1) for data splitting. Central packages used for writing AWESOM as well as training and validating include NetworkX (v.3.4.2), NumPy (v.1.26.4), Optuna (v.4.1.0),

Pandas (v.2.1.4), PyTorch (v.2.5.1), PyTorch Geometric (v.2.6.1), PyTorch Lightning (v.2.5.0) and RDKit (v.2023.3.3). Data analysis and visualization was done in Python (v.3.10.6) using RDKit (v.2023.3.3), matplotlib (v.3.10.0) and seaborn (v.0.13.2).

S2 Data

S2.1 Data preprocessing

The following preprocessing steps were applied to the MetaQSAR database:[1,2] (1) Remove any entries for which an international chemical identifier (InChI) cannot be computed. Remove any entries with identical MetaQSAR molecular identifiers but differing InChIs. (2) Discard compounds containing any chemical element other than H, B, C, N, O, F, Si, P, S, Cl, Br, and I. (3) Discard compounds with molecular mass above 1,000 Da. (4) Discard compounds with fewer than 5 heavy atoms. (5) Remove reactions where a site-of-metabolism (SOM) is labeled as uncertain by the curators of the MetaQSAR database. The uncertainty property is a binary label applied to each recorded SOM. (6) Standardize and canonicalize all molecular structures using a modified version of the ChEMBL Structure Pipeline.[3] This pipeline executes a series of standardization steps, including kekulization, removal of salts, and standardized assignment of charges. The modification removes the separation of charges on the sulfoxide functional group, as it interferes with atom indexation and, consequently, the SOM annotation. (7) Remove all stereochemical information and merge duplicated molecular structures based on their InChIs. (8) Assign positive labels to atoms topologically identical to atoms already labeled as SOM.

Table S1 details the number of reactions and compounds pruned by each preprocessing step. The obtained preprocessed data set consists of 2,331 xenobiotics participating in 5,053

biotransformations.

Table S1: Number of reactions and substrates pruned by each preprocessing step.

**Preprocessing step**	**Number of pruned reactions**	**Number of pruned compound**
Removal of compounds with invalid InChI	16	8
Removal of compounds with unusual chemical elements	152	102
Removal of compounds with molecular mass more than 1,000 Da	28	9
Removal of compounds with less than 5 heavy atoms	50	31
Removal of compounds with uncertain SOMs	397	38
Removal of compounds for which standardization failed	5	4
Removal of duplicate compounds after stripping stereochemical information	619	302

S2.2 Data splitting and analysis

Table S2 contains key figures describing the composition of the data used in this work.

Table S2: Composition of the data set used in this work.

	**Number of substrates**	**Number of heavy atoms**	**Number of SOMs**	**Avg. number of SOMs per substrate**	**Fraction of SOMs among heavy atoms**
whole set	2,331	52,737	5,471	2.35	0.10
training set	1,971	44,642	4,589	2.33	0.10
test set	360	8,095	882	2.45	0.11

Figure S1 and Table S3 present the total number of atoms, the number of SOMs and the proportion of SOMs per atom type in the MetaQSAR data set. The atom types include boron, bromine, chlorine, fluorine, iodine, silicon, carbon, nitrogen, oxygen, and sulfur. The latter four are further categorized by their hybridization state; for instance, "C-SP3" represents an sp^3-hybridized carbon atom.

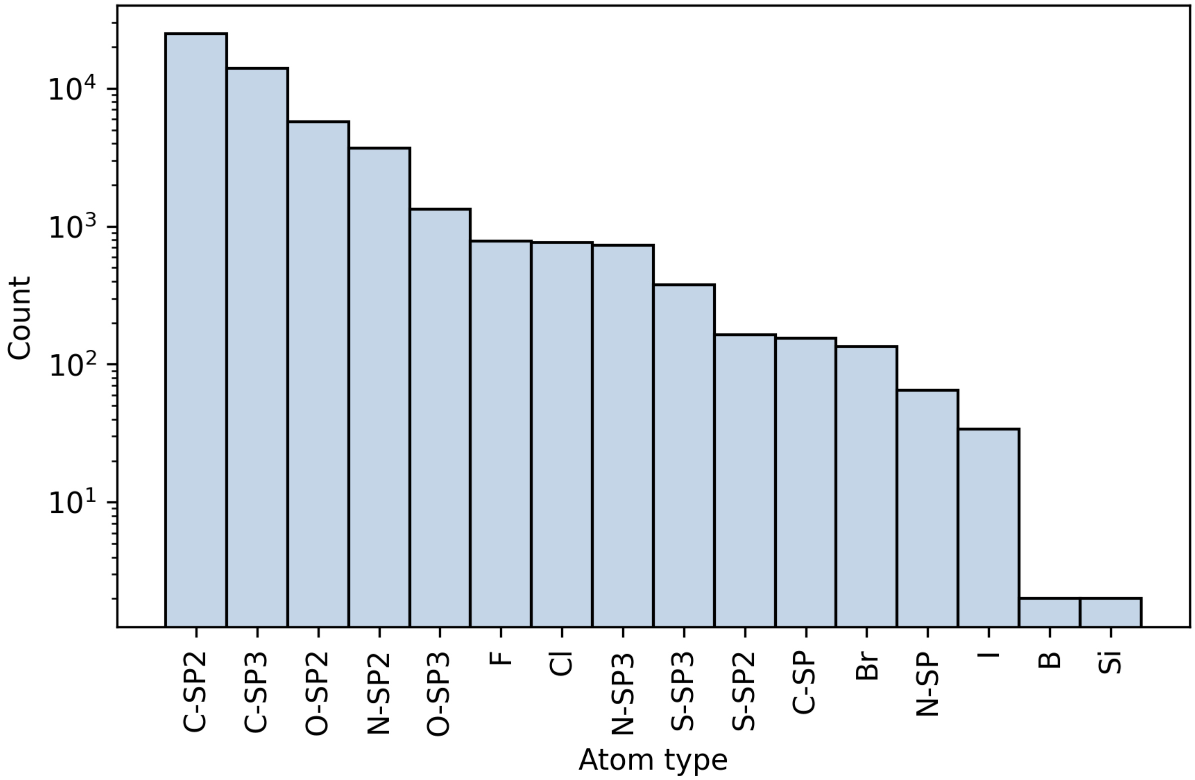

Figure S1: Total number of atoms per atom type in the MetaQSAR data set. Note that the y-axis is scaled logarithmically.

Table S3: Total number of atoms, number of SOMs and proportion of SOMs across atom types in the MetaQSAR data set.

Atom type	Total count	SOM count	SOM ratio [%]
C-SP2	24928	1880	8
C-SP3	13885	1919	14
O-SP2	5698	664	12
N-SP2	3691	421	11
O-SP3	1329	223	17
F	779	0	0
Cl	764	0	0
N-SP3	730	167	23
S-SP3	377	111	29
S-SP2	163	43	26
C-SP	155	35	23
Br	135	0	0
N-SP	65	3	5
I	34	0	0
B-SP2	2	1	50
Si-SP3	2	0	0

S2.3 Data featurization

Depending on the employed architecture (see Section S3), atomic features (node level), bond features (edge level), and molecular features (graph level) were computed using RDKit. The full list of features can be found in Table S4.

Table S4: Atom-, bond-, and molecule-level input features used in this work.

**graph level**	**chemical level**	**feature**	**values**	**dimension**
node	atom	atom type	B, C, N, O, F, Si, P, S, Cl, Br, I	11
edge	bond	bond type	single, double, triple, aromatic	4
		is in a ring	bool	1
		is in a conjugated system	bool	1
graph	molecule	molecular weight	float	1
		logP	float	1
		Labute accessible surface area	float	1
		topological surface area	float	1
		no. hydrogen bond acceptors	integer	1
		no. hydrogen bond donors	integer	1
		no. heavy atoms	integer	1
		no. heteroatoms	integer	1
		fraction of sp^3 hybridized carbon atoms	float	1
		no. rings	integer	1
		no. heterocycles	integer	1
		no. aliphatic carbocycles	integer	1
		no. aliphatic heterocycles	integer	1
		no. aromatic carbocycles	integer	1
		no. aromatic heterocycles	integer	1
		no. saturated carbocycles	integer	1
		no. rotatable bonds	integer	1
		no. amide bonds	integer	1

S3 Model architecture

AWESOM's architecture is fully described in the main body of this work in Section 2.5, and is depicted schematically in Figure 2.

In the initial development phase, we experimented with various graph convolutional operators: MF, GIN, GINE, and GATv2 (see Section S3.4 for a detailed explanation of these operators). Each model was structured into two main components: a convolutional module and a classification module. The convolutional module contained n convolutional layers of size m, interspersed by batch normalization and LeakyReLU activation[4] layers. The classification module begins with two fully connected layer of size s, each followed by batch normalization, LeakyReLU activation, and dropout layers. The dropout probability was set to 0.2. The classification module ends with a final fully connected layer that maps the high-dimensional latent representation to a single logit, which is then passed through the sigmoid function to produce a probabilistic binary output.

All models were trained using the AdamW optimizer,[5] weighted binary cross-entropy loss, and batch size 32. The positive class weight w was optimized so as to achieve the best possible balance between precision and recall, thus maximizing the Matthew's correlation coefficient (MCC), our primary evaluation metric. We searched for the optimal values of n, m, s, and w, as well as the best values for the learning rate and weight decay with the Optuna library[6] through 10-fold cross-validation, assessing each configuration's performance as the average across all 10 validation folds. The initial learning rate was reduced by a factor of 10 after 10 epochs without improvement of the validation loss, and early stopping was applied after 20 epochs without improvement. The number of Optuna trials was set to 20. The hyperparameters and 10-fold CV performance of all models can be found in Table S5.

S3.1 Molecular context pooling

In a subsequent development phase, we incorporated molecular context into the model's architecture with the best-performing graph convolutional operator, identified as GINE. This was achieved by applying additive pooling to the node representations $\{h_1, ..., h_{|V|}\}_i$ of the molecular graph G_i and concatenating the pooled representation to the representation of the individual nodes j constituting G_i. We call this strategy molecular context-pooling.

$$h_j \leftarrow \text{concat}\left(h_j, \sum_{i=1}^{|V|} h_i\right) \tag{1}$$

Context pooling led to the best validation metrics after 20 Optuna trials (Table S5). The resulting model was subsequently fine-tuned for an additional 30 trials.

S3.2 Molecular features

We explored the use of precomputed molecular features as an alternative to context pooling for incorporating molecular context. This was done by adding normalized pre-computed physicochemical properties (see Table S4 for the list of employed features) to the corresponding node embeddings prior to classification. The results can be found in Table S5.

S3.3 Skip connections

To address oversmoothing and enhance the model's ability to learn long-range interactions across the molecular graph, we explored using skip connections. Specifically, we investigated two distinct approaches: DenseNet-inspired skip connections,[7] where each layer within a dense block receives inputs from all preceding layers and outputs to all subsequent layers,

and ResNet-inspired skip connections,[8] where the input of a layer (or block of layers) is directly added to its output. Both strategies were integrated into the general architecture that utilized the best-performing operator, GINE.

Table S5: Optimal hyperparameters of all investigated architectures after hyperparameter optimization (20 Optuna trials). Note that the best performing architecture (GINE + CP) was optimized for another 30 trials to yield the final model, which is fully described in the main body of this work in Section 2.5.

	initial learning rate	weight decay	positive class weight	num. conv. layers	size conv. layers layers	size fully-connected	num. epochs
	$[10^{-6}, 10^{-3}]$	$[10^{-5}, 10^{-2}]$	[2, 3]	[1, 6]	[64, 1024]	[64, 1024]	[0, 500]
MF[1]	5×10^{-6}	8×10^{-4}	2.90	5	314	226	107
GIN	3×10^{-6}	9×10^{-3}	2.29	4	987	135	183
GINE	3×10^{-4}	7×10^{-5}	2.16	3	873	74	46
GATv2[2]	16×10^{-5}	7×10^{-3}	2.09	6	362	146	42
GINE + CP[3]	8×10^{-4}	3×10^{-3}	2.44	6	162	937	43
GINE + MP[4]	7×10^{-4}	7×10^{-3}	2.00	4	64	984	37
GINE + SC1[5]	3×10^{-5}	7×10^{-5}	2.81	5	415	108	60
GINE + SC2[6]	5×10^{-5}	1×10^{-2}	2.01	5	976	92	51

[1] Additional hyperparameter for MF: maximum degree = 5
[2] Additional hyperparameters for GATv2: heads = 4, negative slope = 0.13
[3] CP: context pooling
[4] MP: molecular properties
[5] SC1: skip connections in the style of DenseNet
[6] SC2: skip connections in the style of ResNet

S3.4 Message passing graph convolutional operators

Graph neural networks (GNNs) are a class of neural network designed to work in irregularly structured data such as graphs and point clouds. GNNs operate by propagating information across the graph, allowing nodes to exchange information with their neighbors. This process is called neighborhood aggregation or message passing.[9] With $x_i^{k-1} \in \mathbb{R}^F$ denoting the node features of node i in layer $(k-1)$ and $e_{(i,j)} \in \mathbb{R}^D$ denoting the optional edge features from node i to node j, message-passing graph neural networks can be described as

Table S6: 10-fold cross-validation performance of all investigated architectures after hyperparameter optimization (20 Optuna trials).

	ROC-AUC	PR-AUC	F1	MCC	precision	recall	TOP-2
	[0,1]	[0,1]	[0,1]	[-1,1]	[0,1]	[0,1]	[0,1]
MF	0.83 ± 0.03	0.44 ± 0.07	0.45 ± 0.04	0.38 ± 0.04	0.39 ± 0.03	0.55 ± 0.07	0.74 ± 0.05
GIN	0.85 ± 0.03	0.47 ± 0.06	0.48 ± 0.04	0.42 ± 0.06	0.43 ± 0.06	0.56 ± 0.01	0.72 ± 0.09
GINE	0.85 ± 0.03	0.46 ± 0.05	0.43 ± 0.06	0.37 ± 0.07	0.43 ± 0.07	0.44 ± 0.07	0.76 ± 0.07
GATv2	0.84 ± 0.02	0.46 ± 0.07	0.45 ± 0.05	0.39 ± 0.05	0.46 ± 0.06	0.45 ± 0.07	0.74 ± 0.09
GINE + CP[1]	0.88 ± 0.03	0.50 ± 0.08	0.49 ± 0.05	0.44 ± 0.07	0.42 ± 0.05	0.59 ± 0.12	0.77 ± 0.07
GINE + MP[2]	0.86 ± 0.03	0.50 ± 0.10	0.49 ± 0.11	0.43 ± 0.12	0.49 ± 0.10	0.49 ± 0.12	0.75 ± 0.08
GINE + SC1[3]	0.87 ± 0.02	0.51 ± 0.04	0.48 ± 0.04	0.42 ± 0.04	0.41 ± 0.05	0.58 ± 0.06	0.76 ± 0.06
GINE + SC2[4]	0.87 ± 0.02	0.51 ± 0.07	0.50 ± 0.04	0.44 ± 0.05	0.48 ± 0.05	0.54 ± 0.09	0.82 ± 0.05

[1] CP: context pooling
[2] MP: molecular properties
[3] SC1: skip connections in the style of DenseNet
[4] SC2: skip connections in the style of ResNet

$$x_i^{(k)} = \gamma^{(k)}\left(x_i^{(k-1)}, \bigoplus_{j \in \mathcal{N}(i)} \phi^{(k)}\left(x_i^{(k-1)}, x_j^{(k-1)}, e_{i,j}\right)\right), \tag{2}$$

where $\bigoplus$ denotes a differentiable, permutation invariant function, and γ and ϕ denote differentiable functions such as multi-layer perceptrons.

In this work, we evaluated a range of spatial graph convolutional operators and model architectures, which are presented in the following paragraphs.

S3.4.1 Molecular fingerprints graph convolutional network

The first graph convolutional operator evaluated in our study is derived from the seminal work by Duvenaud *et al.* titled "Convolutional Networks on Graphs for Learning Molecular Fingerprints".[10] The operator is referred to as MF throughout the paper and defined as

$$x_i^{(k)} = W_1^{(deg(i))} x_i^{(k-1)} + W_2^{(deg(i))} \sum_{j \in \mathcal{N}(i)} x_j^{(k-1)}, \tag{3}$$

where W_1 and W_2 are distinct weight matrices for each possible vertex degree.

S3.4.2 Graph isomorphism network

The graph isomorphism network (GIN) operator was designed to enhance the expressive power of graph neural networks by allowing the network to distinguish between graphs that are non-isomorphic, thereby improving performance on tasks requiring precise graph representation.[11] In this work, we tested both the original GIN operator[11] and its modified version that incorporates edges features into the aggregation procedure, GINE,[12] which is defined as

$$x_i^{(k)} = \gamma^{(k)} \left((1+\epsilon) \cdot x_i^{(k-1)} + \sum_{j \in \mathcal{N}(i)} \mathrm{ReLU}(x_j^{(k-1)}, e_{i,j} \right) \tag{4}$$

S3.4.3 Graph attention network

The graph attention network (GAT) operator improves the standard graph neural network operator by using attention to weigh the importance of neighboring nodes during message passing. This allows GATs to selectively focus on relevant neighbors and dynamically adjust their influence, leading to more flexible and discriminative node representations. The GATv2 operator from Brody *et al.*[13] is defined as

$$x_i^{(k)} = \sum_{j \in \mathcal{N}(i) \cup \{i\}} \alpha_{i,j} \Theta_j x_j, \tag{5}$$

where the attention coefficients $\alpha_{i,j}$ are computed as

$$\alpha_{i,j} = \frac{\exp(\mathrm{LeakyReLU}(a_s^\top \Theta_s x_i + a_t^\top \Theta_t x_j + a_e^\top \Theta_e e_{i,j}))}{\sum_{k \in \mathcal{N}(i) \cup \{i\}} \exp(\mathrm{LeakyReLU}(a_s^\top \Theta_s x_i + a_t^\top \Theta_t x_k + a_e^\top \Theta_e e_{i,k}))} \tag{6}$$

Note that if the graph is not bipartite, $\Theta_s = \Theta_t$.

S4 Impact of uncertainty estimation on the runtime

Runtime analysis was conducted on the test set (360 compounds). The results are shown in Table S7. The total runtime is divided into the time required for loading and preprocessing the data, applying the model (*i.e.*, predicting SOMs) and computing and logging the test results and performance metrics. Running the model as an ensemble of 10 individual models to estimate uncertainty *via* deep ensembling increases the prediction step's runtime by approximately four-fold. However, the overall runtime remains primarily influenced by data loading and preprocessing, and logging the results and performance metrics —steps that are unaffected by the inclusion of uncertainty estimation. Consequently, we consider the additional runtime required for uncertainty estimation negligible, with the benefits of obtaining these estimates far outweighing the minor increase in runtime.

Table S7: Runtime impact of uncertainty estimation.

elapsed time [s]	total	data loading & preprocessing	predicting SOMs	computing metrics & logging results
single model (no uncertainty estimation)	13.2 ± 0.6	3.8 ± 0.3	0.52 ± 0.03	8.8 ± 0.4
ensemble (uncertainty estimation)	14.3 ± 0.9	3.8 ± 0.4	1.9 ± 0.4	8.7 ± 0.4

[1] Runtime analysis conducted on a data set of 360 compounds comprising 8,095 heavy atoms, executed on a system equipped with an AMD Ryzen 9 7950X 16-Core CPU and an NVIDIA GeForce RTX 4090 GPU. The numbers correspond to the total runtimes for all 360 compounds.
[2] Mean and standard deviation estimated over 100 runs.

S5 Controlled studies for understanding epistemic and aleatoric uncertainties: predicted SOM-probabilities per atom type

To validate our uncertainty decomposition strategy in the absence of ground-truth uncertainty labels, we conducted two experiments using synthetic data: one probing epistemic uncertainty by removing all halogen-containing molecules from the training set ("halogen-agnostic" model), and another targeting aleatoric uncertainty by injecting label noise into halogen-labeled samples ("noise-injected" model). The main findings of this study are presented in the main body of this work in Section 3.3. Figure S2 shows boxplots of the predicted SOM-probabilities per atom type for AWESOM, the halogen-agnostic and the noise-injected models.

The predicted SOM probabilities align well with our expectations. In the baseline scenario, halogen atoms are consistently assigned probabilities close to zero, reflecting their absence from the SOM-labeled training data. In contrast, non-halogen atom types exhibit a broader range of probabilities between zero and one, depending on their chemical environment. In the halogen-agnostic and noise-injected models, changes are observed only in the predicted

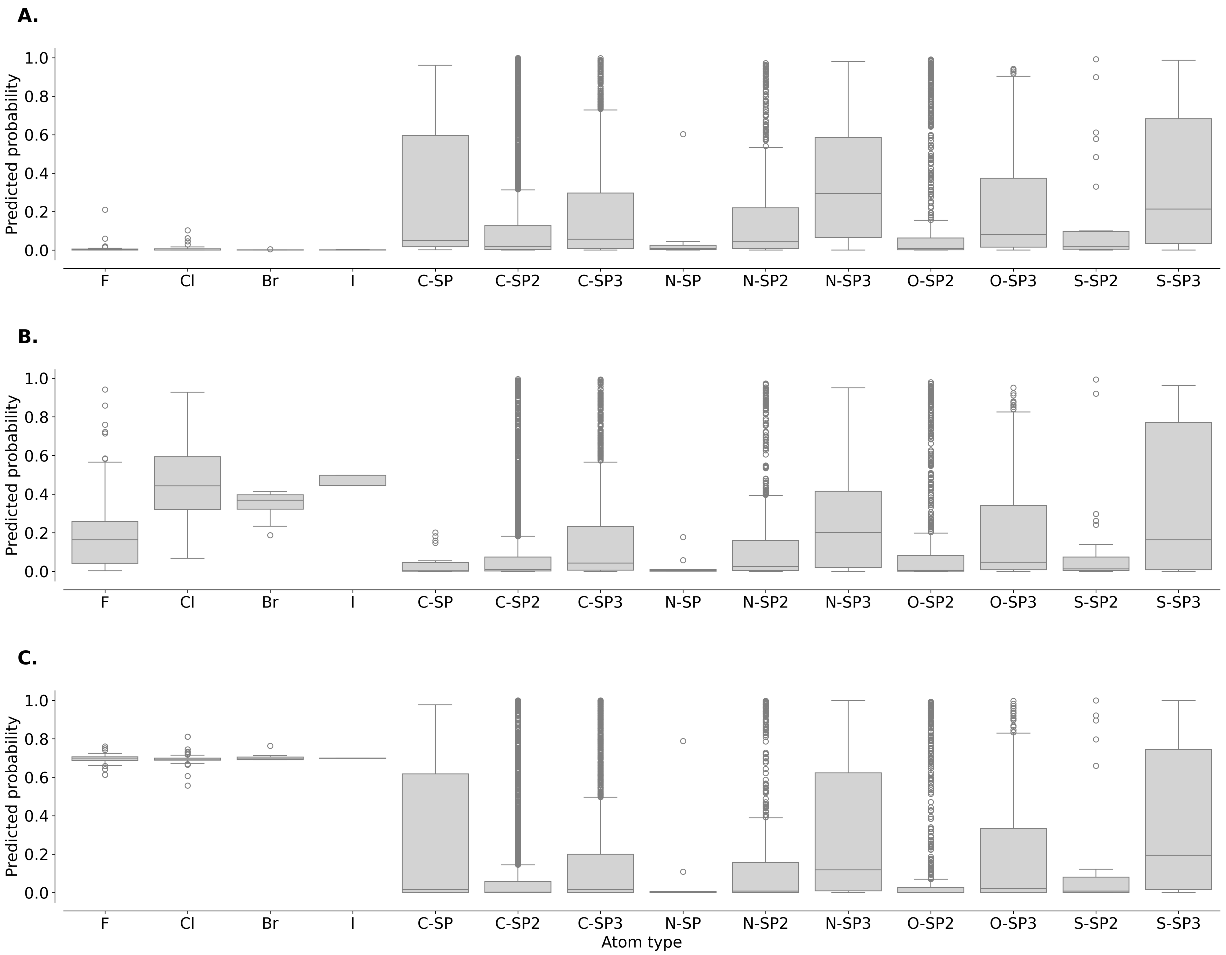

Figure S2: Predicted SOM-probabilities per atom type. **A)** aweSOM (baseline), **B)** halogen-agnostic, **C)** noise-injected.

probabilities for halogen atoms; predictions for other atom types remain largely stable, aside from minor variation due to experimental variability. In the halogen-agnostic model, the absence of relevant training data leads to less reliable predictions for halogens, manifesting as broader probability distributions. Conversely, in the noise-injected model, the predicted probabilities for halogen atoms are more tightly distributed but shifted toward higher values which are consistent with the artificially introduced label noise.

The presence of outliers in the predicted probabilities for halogen atoms in the baseline scenario prompts further analysis. We believe them to arise from the combined effects of the model architecture, the diversity of atomic environments, and the nature of the training objective.

aweSOM model uses learned embeddings that encode not only atom-level features but also information from the atom's local chemical environment (determined by the receptive field of four convolutional layers) as well as global molecular context via the context pooling module. These design choices help the model capture complex chemical patterns critical for accurate SOM prediction. However, they also mean that each atom is represented in the context of a highly specific substructure, making most atomic environments effectively unique, except in cases of molecular symmetry. This uniqueness prevents the model from memorizing fixed relationships between atom types and SOM labels. Instead, it must generalize by placing each atom in a high-dimensional embedding space and predicting its SOM probability based on its position relative to similar (but not identical) examples from the training set. Because this embedding space is not perfectly smooth, small differences in structure or context can lead to variability in predicted probabilities, even among atoms of the same type.

This variability is further influenced by the training objective. The model is trained using binary cross-entropy loss, which encourages correct classification rather than precise probability calibration. It only requires that positive examples receive probabilities above 0.5 and

negatives below 0.5; there is no incentive to push predictions for negative atoms all the way to zero. As a result, even atom types that are consistently labeled as negative (e.g., halogens) can still exhibit some variation in their predicted SOM probabilities, leading to the observed outliers. In summary, the combination of context-sensitive embeddings, generalization across chemically diverse inputs, and a classification-oriented loss function explains the observed spread and occasional outliers in the prediction distributions.

References

(1) Pedretti, A.; Mazzolari, A.; Vistoli, G.; Testa, B. MetaQSAR: An Integrated Database Engine to Manage and Analyze Metabolic Data. *J. Med. Chem.* **2018**, *61*, 1019–1030.

(2) Pedretti, A.; Mazzolari, A.; Vistoli, G.; Testa, B. MetaQSAR Database (snapshot from October 2023).

(3) Bento, A. P.; Hersey, A.; Félix, E.; Landrum, G.; Gaulton, A.; Atkinson, F.; Bellis, L. J.; Veij, M. D.; Leach, A. R. An Open Source Chemical Structure Curation Pipeline Using RDKit. *J. Cheminf.* **2020**, *12*, 51.

(4) Maas, A. L.; Hannun, A. Y.; Ng, A. Y. Rectifier Nonlinearities Improve Neural Network Acoustic Models. *Proceedings of the 30th International Conference on Machine Learning, Atlanta, United States of America, June 16-21* **2013**,

(5) Loshchilov, I.; Hutter, F. Decoupled Weight Decay Regularization. *Proceedings of The 7th International Conference on Learning Representations, New Orleans, United States of America, May 6-9* **2019**, DOI: 10.48550/arXiv.1711.05101.

(6) Akiba, T.; Sano, S.; Yanase, T.; Ohta, T.; Koyama, M. Optuna: A Next-Generation Hyperparameter Optimization Framework. *Proceedings of the 25th ACM SIGKDD*

International Conference on Knowledge Discovery and Data Mining, Anchorage, United States of America, August 4-8 **2019**, 2623–2631.

(7) Huang, G.; Liu, Z.; van der Maaten, L.; Weinberger, K. Q. Densely Connected Convolutional Networks. 2018; https://arxiv.org/abs/1608.06993.

(8) He, K.; Zhang, X.; Ren, S.; Sun, J. Deep Residual Learning for Image Recognition. 2015; https://arxiv.org/abs/1512.03385.

(9) Fey, M.; Lenssen, J. E. Fast Graph Representation Learning with PyTorch Geometric. 2019; https://arxiv.org/abs/1903.02428.

(10) Duvenaud, D.; Maclaurin, D.; Aguilera-Iparraguirre, J.; Gomez-Bombarelli, R.; Hirzel, T.; Aspuru-Guzik, A.; Adams, R. P. Convolutional Networks on Graphs for Learning Molecular Fingerprints. *Proceedings of the 28th International Conference on Neural Information Processing Systems, Montreal, Canada, December 8-13* **2015**, *2*, 2224–2232.

(11) Xu, K.; Hu, W.; Leskovec, J.; Jegelka, S. How Powerful are Graph Neural Networks? 2019; https://arxiv.org/abs/1810.00826.

(12) Hu, W.; Liu, B.; Gomes, J.; Zitnik, M.; Liang, P.; Pande, V.; Leskovec, J. Strategies for Pre-training Graph Neural Networks. *Proceedings of the 8th International Conference on Learning Representations, Addis Ababa, Ethiopia, April 26-30* **2020**, DOI: 10.48550/arXiv.1905.12265.

(13) Brody, S.; Alon, U.; Yahav, E. How Attentive are Graph Attention Networks? 2022; https://arxiv.org/abs/2105.14491.
